# Behningiidae and Potamanthidae (Insecta, Ephemeroptera) in Thailand

**DOI:** 10.3897/zookeys.1067.72779

**Published:** 2021-10-29

**Authors:** Sedtawut Kwanboon, Michel Sartori, Boonsatien Boonsoong

**Affiliations:** 1 Animal Systematics and Ecology Speciality Research Unit (ASESRU), Department of Zoology, Faculty of Science, Kasetsart University, Bangkok 10900, Thailand Kasetsart University Bangkok Thailand; 2 Museum of Zoology, Palais de Rumine, Place Riponne 6, CH-1005 Lausanne, Switzerland Museum of Zoology Lausanne Switzerland; 3 University of Lausanne (UNIL), Department of Ecology and Evolution, CH-1015 Lausanne, Switzerland University of Lausanne Lausanne Switzerland

**Keywords:** Biodiversity, COI, egg, mayfly, new record

## Abstract

The aim of this study is to review the family Behningiidae and Potamanthidae in Thailand. Two genera and three species of Behningiidae are recognised: *Protobehningiamerga* Peters & Gillies, 1991, *Behningiabaei* McCafferty & Jacobus, 2006, and *Behningianujiangensis* Zhou & Bisset, 2019, which is newly reported from Thailand. The egg structure of *B.nujiangensis* is described for the first time using scanning electron microscopy. The larva of *P.merga* is redescribed and its distribution is expended northward with a new record from Chiang Mai province. Two genera and five species of Potamanthidae are identified: *Potamanthusformosus* Eaton, 1892, *Rhoenanthusmagnificus* Ulmer, 1920 (new record for Thailand), *Rhoenanthusobscurus* Navás, 1922, *Rhoenanthusdistafurcus* Bae & McCafferty, 1991, and *Rhoenanthusspeciosus* Eaton, 1881. Our morphological evidence is supported by COI data for the family Potamanthidae. Diagnostic characters, distributions, and keys are presented for the larvae of all known species of Thai behningiid and potamanthid mayflies.

## Introduction

The Behningiidae is a small mayﬂy family represented by three extant genera (*Behningia* Lestage, 1930, *Dolania* Edmunds & Traver, 1959, and *Protobehningia* Tshernova, 1960) and one fossil genus (*Archaeobehningia* Tshernova, 1977) (Hubbard 1994). Members of the Behningiidae are known as tuskless burrowing mayflies for their sand-dwelling behaviour (Miller et al. 2018). To date, seven species have been described, and three of them have been documented in the Oriental region (two species from Thailand and one species from China) (Zhou et al. 2019). *Behningiabaei* McCafferty & Jacobus, 2006 and *Protobehningiamerga* Peters & Gillies, 1991 have been described from Thailand and to date have only been reported from the type localities (Peters and Gillies 1991; Parnrong et al. 2002; McCafferty and Jacobus 2006). Only larval exuviae of *P.merga* are known (Peters and Gillies 1991).

The family Potamanthidae is widely distributed throughout the Holarctic and Oriental regions and accounts for 25 species worldwide. In Southeast Asia, seven species in two genera and four subgenera have been reported (Nguyen and Bae 2004). Bae and McCafferty (1991) recognised subgenera within *Potamanthus* and *Rhoenanthus*, and we followed this classification in the present study. The known species in Thailand are Potamanthus (Potamanthodes) formosus Eaton, 1982, Rhoenanthus (Rhoenanthus) distafurcus Bae & McCafferty, 1991, Rhoenanthus (Rhoenanthus) speciosus Eaton, 1881, and Rhoenanthus (Potamanthindus) obscurus Navás, 1922 (Bae and McCafferty 1991; Parnrong et al. 2002).

In this study, we review the species of Behningiidae and Potamanthidae in Thailand, and we provide the first records of *B.nujiangensis* Zhou & Bisset, 2019 and *R.magnificus* Ulmer, 1920. We also redescribe the larva of *P.merga*, and we present the first description of the egg structure of *B.nujiangensis*. A distribution map of Thai behningiid and potamanthid mayflies is also provided.

## Materials and methods

The specimens were collected from streams and rivers in Thailand and were preserved in absolute ethanol. Measurements (in mm) and photographs were taken using a Nikon SMZ800 and ZEISS Stemi 305 stereoscopic microscope. For scanning electron microscopy (SEM), specimens (head, legs, labrum, labium, labial palp, glossa, paraglossa, and eggs) were dried in a critical point dryer (CPD7501) and coated with gold (Sputter Coater SC7620). The specimens were observed and photographed with an FEI Quanta 450 SEM. The final plates were prepared with Adobe Photoshop CC 2020. The material is deposited in the collection of the Zoological Museum at Kasetsart University in Bangkok, Thailand (**ZMKU**). The distribution map was constructed using the Simple Mapper website (http://www.simplemappr.net) and GPS coordinates.

### Molecular methods

Each specimen was dissected for DNA extraction using a genomic DNA purification kit (NucleoSpin, Macherey-Nagel, Germany) following the manufacturer’s protocol. A fragment of the mitochondrial cytochrome oxidase I (COI) was amplified using the primers LCO1490 and HCO2198 (Folmer et al. 1994). The polymerase chain reaction (PCR) conditions and procedure were as described by Gattolliat et al. (2015). The PCR products were purified using a Gel and PCR Clean-up Kit (NucleoSpin, Macherey-Nagel, Germany) and were sequenced by ATGC Co., Ltd (Thailand). The Kimura-2-parameter distances were calculated using the MEGA X program (Kumar et al. 2018). A phylogenetic tree was analysed by the maximum likelihood (ML) method and the Tamura 3-parameter protocol was performed with MEGA X using the likelihood-ratchet method with 1,000 bootstrap replicates. Nucleotide sequences obtained in this study have been deposited in the GenBank database (Table 1). Other analysed mayfly sequences, obtained from the Barcode of Life Data System (BOLD), were *Dolaniaamericana* (**BIT011-04**), *Potamanthusformosus* (**THMAY125-12**), and Rhoenanthuscf.magnificus (**THMAY127-12, THMAY128-12**).

**Table 1.** List of the sequenced specimens.

Species	Code	Collection locality	Collector	Date	GenBank accession number
*Protobehningiamerga*	PM01CM	Chiang Mai	B. Boonsoong	13 Nov 2020	MW792224
*Potamanthusformosus*	PF01NA	Nan	B. Boonsoong	28 Nov 2020	MZ453438
	PF02KN	Kanchanaburi	S. Kwanboon	11 Jul 2019	MZ453439
	PF03CR	Chiang Rai	S. Kwanboon	6 Mar 2021	MZ436659
	PF04CR	Chiang Rai	S. Kwanboon	5 Mar 2021	MZ436660
*Rhoenanthusmagnificus*	RM01NA	Nan	S. Kwanboon	10 Mar 2018	MZ436661
	RM04NA	Nan	B. Boonsoong	28 Nov 2020	MZ436662
	RM05CR	Chiang Rai	S. Kwanboon	6 Mar 2021	MZ436663
	RM06CR	Chiang Rai	S. Kwanboon	7 Mar 2021	MZ436664
*R.obscurus*	RO02FCM	Chiang Mai	S. Kwanboon	15 Nov 2020	MZ436665
	ROO7CM	Chiang Mai	S. Kwanboon	15 Nov 2020	MZ436666
*R.distafurcus*	RD01NA	Nan	B. Boonsoong	28 Nov 2020	MZ436667
	RD02NA	Nan	B. Boonsoong	28 Nov 2020	MZ436668
	RD03KN	Kanchanaburi	B. Boonsoong	15 Oct 2015	MZ436669
	RD04RB	Ratchaburi	B. Boonsoong	19 Apr 2016	MZ436670

## Taxonomy

**Order Ephemeroptera Hyatt & Arms, 1891**

**Family Behningiidae Motas & Bacesco, 1937**

### Genus *Behningia* Lestage, 1930

#### Behningia baei

Taxon classificationAnimaliaEphemeropteraBehningiidae

McCafferty & Jacobus, 2006

CBB5AF64-671B-512F-A1A3-B7BD8DE16789

Figures 7 , 9

##### Materials examined.

None.

##### Diagnosis.

The larvae of *Behningiabaei* McCafferty & Jacobus, 2006 can be distinguished from other *Behningia* species based on the following characteristics: i) labrum deeply emarginate in a V or U shape at anteromedian margin, ii) labial palp I without concavity on outer margin, iii) labial palp II less than 50% length of labial palp III, iv) tarsus of foreleg as long as tibia and v) coxa of hind leg less than 60% as long as femur.

##### Distribution.

Phitsanulok province.

##### Remark.

The larvae of *B.baei* were originally described by McCafferty and Jacobus (2006) and collected from Phitsanulok province (Thailand). In this study, we attempted to collect specimens at the type locality (Klong Nam Kub, Ban Khok Phakwan), but no specimens were found during our fieldwork. However, the habitat of the type locality of *B.baei* is suitable for behningiid larvae, consisting of wadeable, widely flooded rivers with fine sandy bottoms and braided channels (Fig. 7).

#### Behningia nujiangensis

Taxon classificationAnimaliaEphemeropteraBehningiidae

Zhou & Bisset, 2019

2700A301-A420-5238-BE15-10DB500371F4

**Figure 1.**
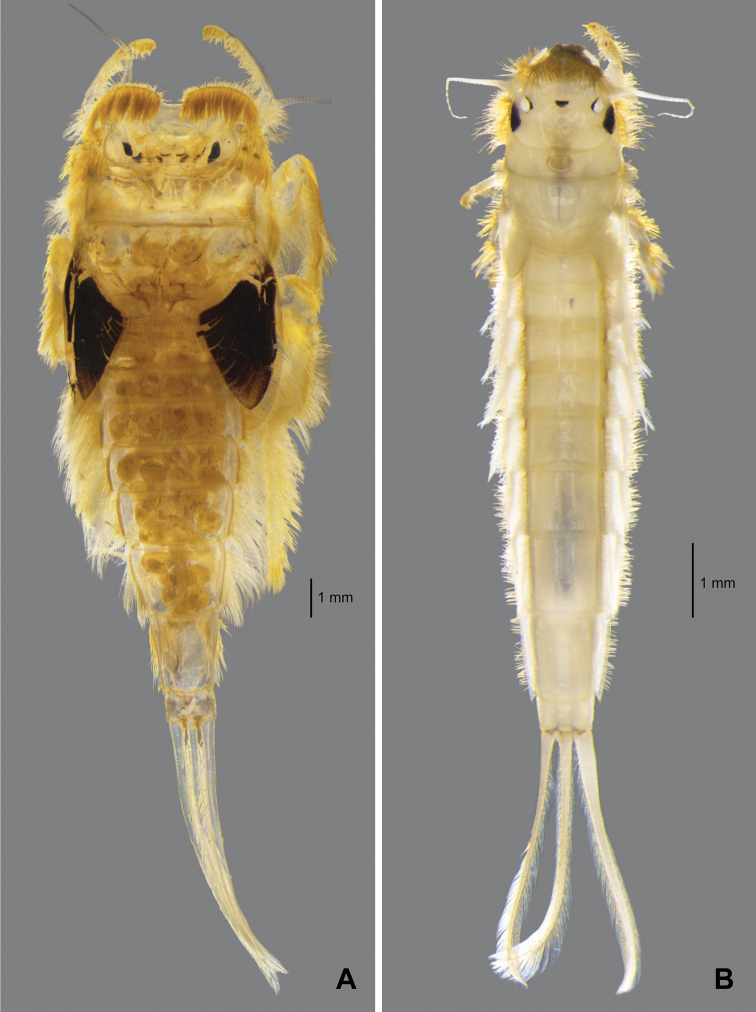
Habitus of larvae **A***Behningianujiangensis* Zhou & Bisset, 2019 **B***Protobehningiamerga* Peters & Gillies, 1991.

**Figure 2.**
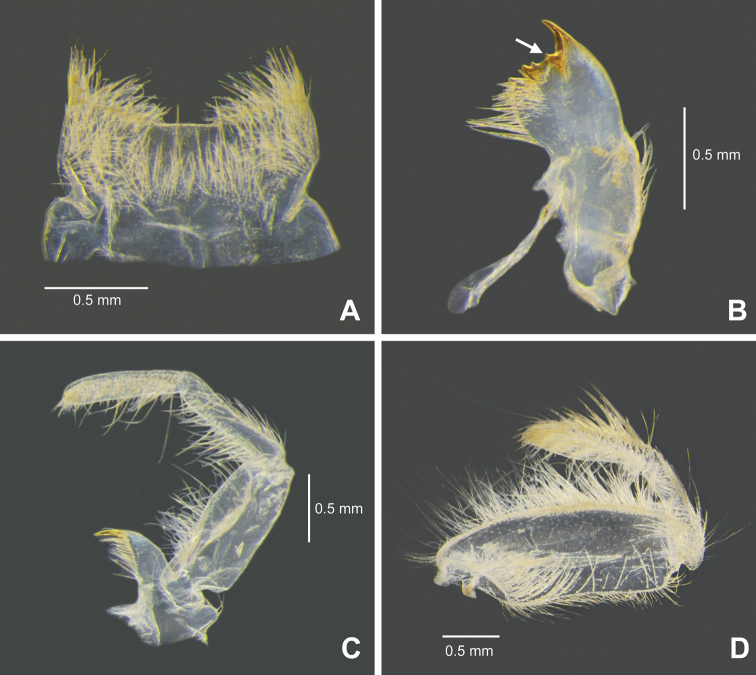
*Behningianujiangensis* Zhou & Bisset, 2019, larval morphology **A** labrum **B** left mandible (ventral view, arrow indicated small spine) **C** left maxilla (ventral view) **D** left labial palp (ventral view).

**Figure 3.**
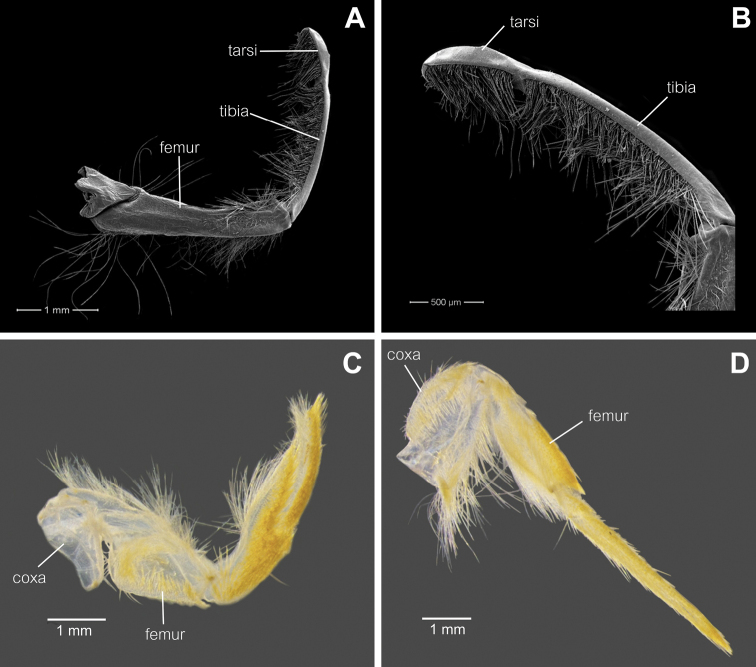
*Behningianujiangensis* Zhou & Bisset, 2019, larval morphology **A** foreleg **B** closer view of tibia and tarsi of foreleg **C** middle leg **D** hind leg.

**Figure 4.**
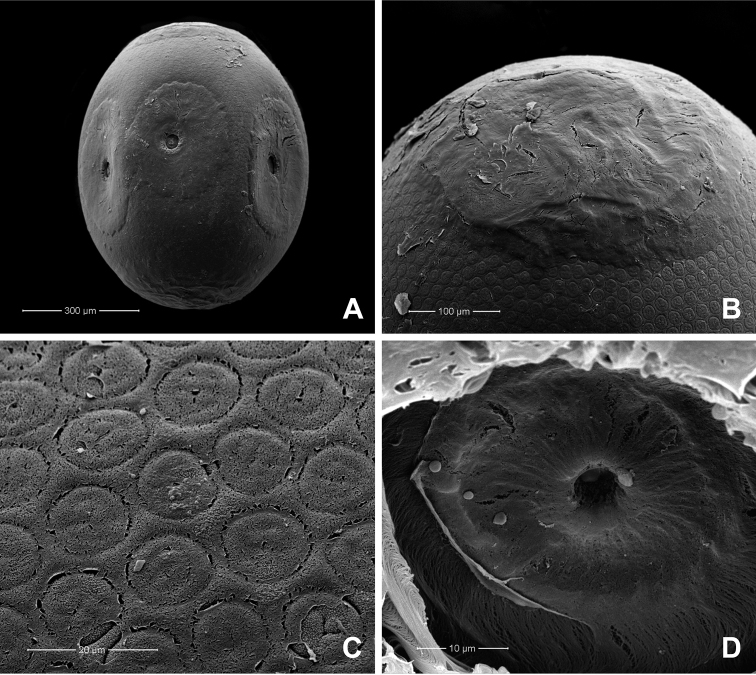
*Behningianujiangensis* Zhou & Bisset, 2019, SEMs of egg structures **A** general outline of egg **B** polar cap **C** chorion surface **D** micropyle.

Figures 1A , 2 , 3 , 4 , 9

##### Materials examined.

2 mature larvae, Thailand, Chiang Mai province, Mae Tang district, Tard Luang Waterfall, 19°01'27.5"N, 98°51'17.1"E, 18.IX.2011, P. Sritipsak leg. deposited in ZMKU.

##### Diagnosis.

The larvae of *Behningianujiangensis* Zhou & Bisset, 2019 can be separated from those of other *Behningia* species based on the following characteristics: i) labrum shallowly emarginate at anteromedian margin (Fig. 2A), ii) molar areas of mandible with a small apical spine (Fig. 2B), iii) galea-lacinia of maxilla elongated and slender (Fig. 2C), iv) labial palp 3-segmented, segment II as long as segment III (Fig. 2D), v) tarsus of forelegs about 40% the length of tibia (Fig. 3A, B), vi) middle and hind legs with coxa as long as femur (Fig. 3C, D).

**Egg** (dissected from mature larva). Length 1.62–1.73 mm, width 1.09–1.26 mm (*n* = 13); oval (Fig. 4A); with massive amounts of fibrous adhesive material localised at the polar and equatorial regions of the egg (Fig. 4B); chorion densely and finely punctutated, with a weakly developed pentagonal reticulation, circular in shape and convex in the middle (Fig. 4C); funnelform micropyle in the centre of circular accumulations of adhesive material only at the equatorial zone (Fig. 4D).

##### Distribution.

Chiang Mai province.

##### Remark.

The larvae of *B.nujiangensis* were originally described by Zhou et al. (2019) and collected from China (Nujiang river, Yunnan province, upper Salween river). In Thailand, the samples were collected from the Tard Luang waterfall (fine sandy habitat) in 2011, and specimens were deposited but only identified to the genus level (Dr. Akekawat Vitheepradit, Department of Entomology, Kasetsart University). In this study, we re-examined and identified the specimens. We attempted to collect specimens from the same microhabitat near the Tard Luang waterfall; however, unfortunately, no specimens were found.

###### Genus *Protobehningia* Tshernova & Bajkova, 1960

#### Protobehningia merga

Taxon classificationAnimaliaEphemeroptera

Peters & Gillies, 1991

E47AD7D9-ECE5-56F7-863F-E00403810630

**Figure 5.**
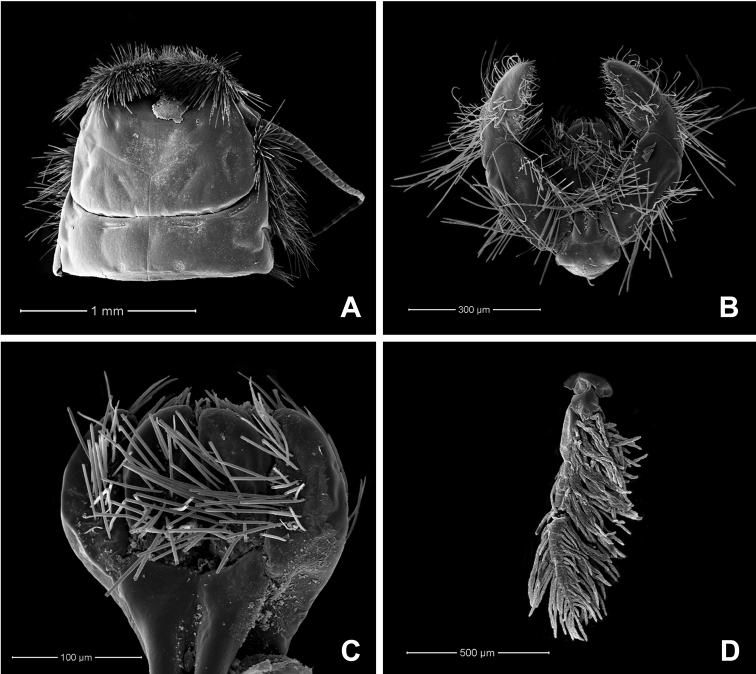
*Protobehningiamerga* Peters & Gillies, 1991, larval morphology **A** overview of head **B** labium (ventral view) **C** glossa & paraglossae (ventral view) **D** gill I.

**Figure 6.**
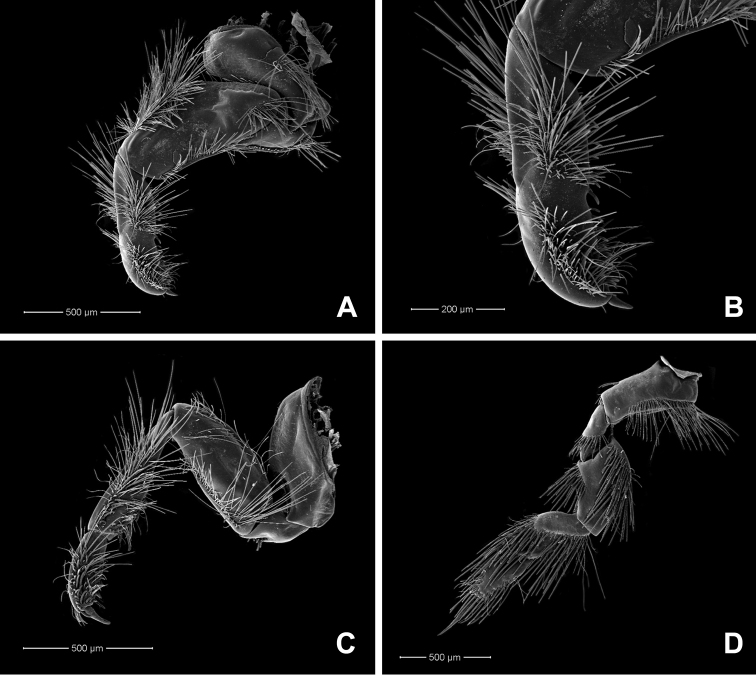
*Protobehningiamerga* Peters & Gillies, 1991, larval morphology **A** foreleg **B** closer view of tibia and tarsi of foreleg **C** middle leg **D** hind leg.

Figures 1B , 5 , 6 , 8 , 9

##### Materials examined.

2 larvae, deposited in ZMKU, Thailand, Chiang Mai province, Mae Chaem district, Mae Chaem river, 18°30'46.0"N 98°21'22.6"E, 475 m, 5.X.2019, B. Boonsoong leg., 1 larva, same data, 13.XI.2020, B. Boonsoong leg. (ZMKU).

##### Re-description of larva.

**Larva** (in alcohol, Fig. 1B) Body length 7.2 mm without cerci; cerci 2.7 mm. Body pale yellowish.

**Head.** Anterior margin not projecting, front with densely short goldish setae standing out on the head (Fig. 1B, 5A). Black eyes on dorsolateral margin; ocelli almost white, inner margin of ocelli black in front of compound eye. Antennae at lateral margin of head. Labium extending the entire anterior margin of head, with long setae; labial palp 3-segmented, surface of labial palp covered with rows of long blunt setae, base of second palp segment with the longest setae, first segment longer than other segments (Fig. 5B), glossae and paraglossae with numerous (>20) setae (Fig. 5C). Left mandible and right mandible strong and dentated, mostly similar to *P.asiatica*. Maxillary palpi 3-segmented, maxilla base extending, apex narrow with terminal tooth.

**Thorax.** Colour pale yellowish. Forelegs flattened, with large broad coxae, flat femur, small claws (Fig. 6A), tarsi fused with tibiae (Fig. 6B), outer margin with long row of setae, short setae present at inner margin. Midleg and hindleg tarsus and tibia not fused (Fig. 6C, D), hindleg with strong claw, curved, thorn-like in shape.

**Abdomen.** Similar in colour to head and thorax, abdominal segments elongated and convex, with short straight setae at lateral margin, lateral margin of abdominal segment I–IX with flat projections spine-like in shape. Gill present on segment I–VII, plumose shape, first gill filament single (Fig. 5D); gills II–VII double, upper branch of each gill shorter than lower one. Three caudal filaments fringed with short pale setae, length of median filament as long as lateral filaments.

##### Diagnosis.

The larvae of *Protobehningiamerga* Peters & Gillies 1991 can be distinguished from those of *P.asiatica* based on the following characteristics: i) glossae and paraglossae with more than 20 setae on the ventral surface, ii) maxillary palp segment II 2/3 the length of segment I, each maxillary palp segment completely divided, but segments II and III indistinct (Peters and Gillies 1991).

##### Distribution.

Kanchanaburi and Chiang Mai provinces.

##### Biological aspects.

In general, the larvae of behningiid mayflies are rarely collected. In this study, the larvae (middle instar) were found in October (turbidity from flooding, Fig. 8A) and November in a river in Chaing Mai province, whereas Peters and Gillies (1991) found the exuviae and imago during December in Kanchanaburi province (western Thailand). The specimens were collected from the Mae Chaem river, which is submontane and bordered by farmland and residential areas (Fig. 8A). The substrates were covered with fine- and coarse-grained sand (Fig. 8C). The larvae of *P.merga* were collected using an aquatic net in a fine sandy habitat, where the depth of the sandy bottom was more than 50 cm and near the littoral zone (Fig. 8B, D). The larvae were usually found together with those of the oligoneuriid mayfly, *Chromarcysmagnifica* Navás, 1932 and the gomphid dragonfly, *Paragomphuscapricornis* Förster, 1914.

##### Remarks.

Only two species of *Protobehningia* are known in the world: *Protobehningiaasiatica* Tshernova & Bajkova 1960 and *Protobehningiamerga* Peters & Gillies 1991. Peters and Gillies (1991) used larval exuviae of *P.merga* for comparison with *P.asiatica*, but they did not give a more detailed description of the larval stage. The labium structures of our specimens are similar to those of the larval exuviae described by Peters and Gillies (1991). Our new record also expands the geographic distribution of *P.merga* to northern Thailand.

### Key to genera and species of Behningiidae in Thailand

(adapted from Zhou et al. 2019)

**Table:** 

1	Tarsi of forelegs not fused to tibiae (Fig. 3A); tibiae of hind legs reduced (Fig. 3D)	***Behningia***, **2**
–	Tarsi of forelegs fused to tibiae (Fig. 6B); tibiae of hind legs not reduced (Fig. 6D); glossae and paraglossae with more than 20 long stout setae on the ventral surface (Fig. 5C)	***Protobehningia* , *P.merga***
2	Medio-anterior emargination of labrum deep (McCafferty and Jacobus 2006, fig. 1); coxa of hind leg less than 60% as long as femur (McCafferty and Jacobus 2006, fig. 7)	***Behningiabaei***
–	Medio-anterior emargination of labrum very shallow (Fig. 2A); coxa of hind leg as long as femur (Fig. 6D)	***Behningianujiangensis***


**Figure 7.**
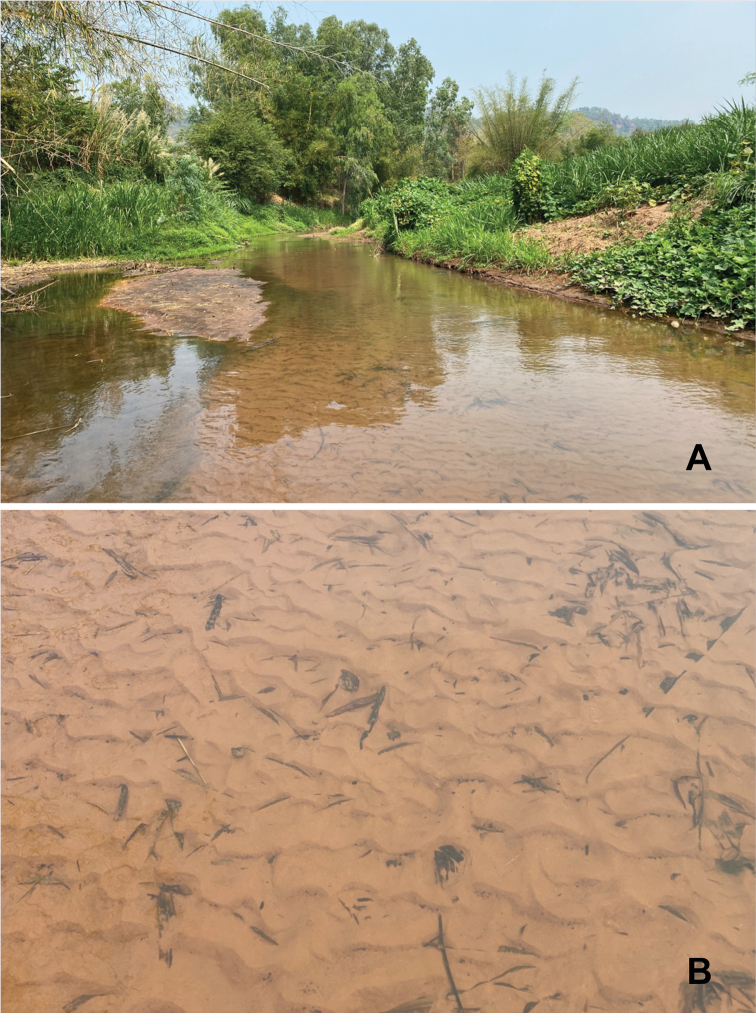
Habitats of *Behningiabaei* McCafferty & Jacobus, 2006 larva **A** Klong Nam Kub stream (March 2021) **B** microhabitat.

**Figure 8.**
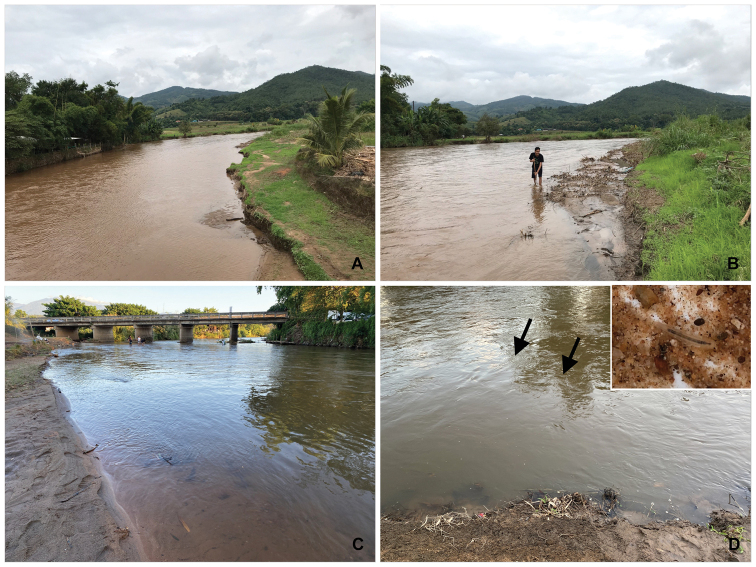
Habitats of *Protobehningiamerga* Peters & Gillies, 1991 larva **A** Mae Chaem wadeable river (October 2019) **B** sampling method **C** sandy bottom river (November 2020) **D** microhabitat.

#### Family Potamanthidae Klapalek,1909

**Genus *Potamanthus* Pictet, 1843**

**SubgenusPotamanthodes Ulmer, 1920**

##### Potamanthus (Potamanthodes) formosus

Taxon classificationAnimaliaEphemeropteraPotamanthidae

Eaton, 1892

30F70820-B88D-5541-AC88-FEC3BF612840

Figures 10A , 11A , 14 , 15

###### Materials examined.

1 larva, Thailand, Chanthaburi province, Makham district, Ban Pa Rim Tarn homestay, 12°51'00.0"N, 102°12'17.1"E, 5.X.2019, B. Boonsoong leg. (ZMKU); 2 larvae, Kanchanaburi province, Huai Pak Kok, 14°39'34.4"N, 98°32'02.3"E, 175 m, 11.VII.2019, S. Kwanboon leg. (ZMKU); 2 larvae, Chiang Rai province, Huai Kang Pla waterfall, 20°05'21.6"N, 99°46'47.8"E, 519 m, 5.III.2021, S. Kwanboon leg. (ZMKU); 4 larvae, Chiang Rai province, Klong Mae Salong, 20°09'52.0"N, 99°40'06.8"E, 6.III.2021, S. Kwanboon leg. (ZMKU); 1 larva, Nan province, Ban Ratsadonsamakkhi, 18°52'23.4"N, 100°49'54.1"E, 59 m, 28.XI.2020, B. Boonsoong leg.

###### Diagnosis.

The larvae of *Potamanthusformosus* Eaton, 1892 can be distinguished from those of other Potamanthus (Potamanthodes) species based on the following characteristics: i) dorsal forefemora with simple stout setae (Fig. 11A), ii) a subapical cluster of setae on the foretibia, iii) short mandibular tusk (0.10–0.23× length of the head) (Fig. 10A), and iv) relatively small body length.

**Figure 9.**
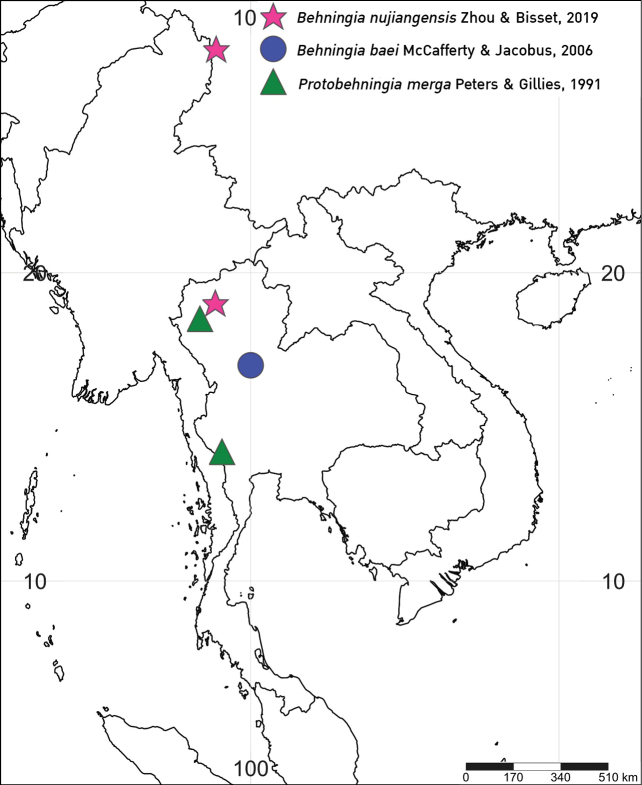
Distribution map of the family Behningiidae in Thailand.

###### Distribution.

Chanthaburi, Kanchanaburi, Nan, and Chiang Rai provinces.

###### Remark.

The adult of *P.formosus* was described by Eaton (1892) based on materials from Myanmar. Imanishi (1940) described the species *Potamanthuskamonis* based on imaginal and larval materials from Japan, and *P.kamonis* was synonymized with *P.formosus* by Ueno (1969). *Potamanthusformosus* is widely distributed in East Asia and Southeast Asia (China, Japan, South Korea, Malaysia, Vietnam, Myanmar, and Thailand). In the present study, the specimens were found in eastern, western, and northern Thailand, so *P.formosus* is the most widespread potamanthid in Thailand (Fig. 14).

**Figure 10.**
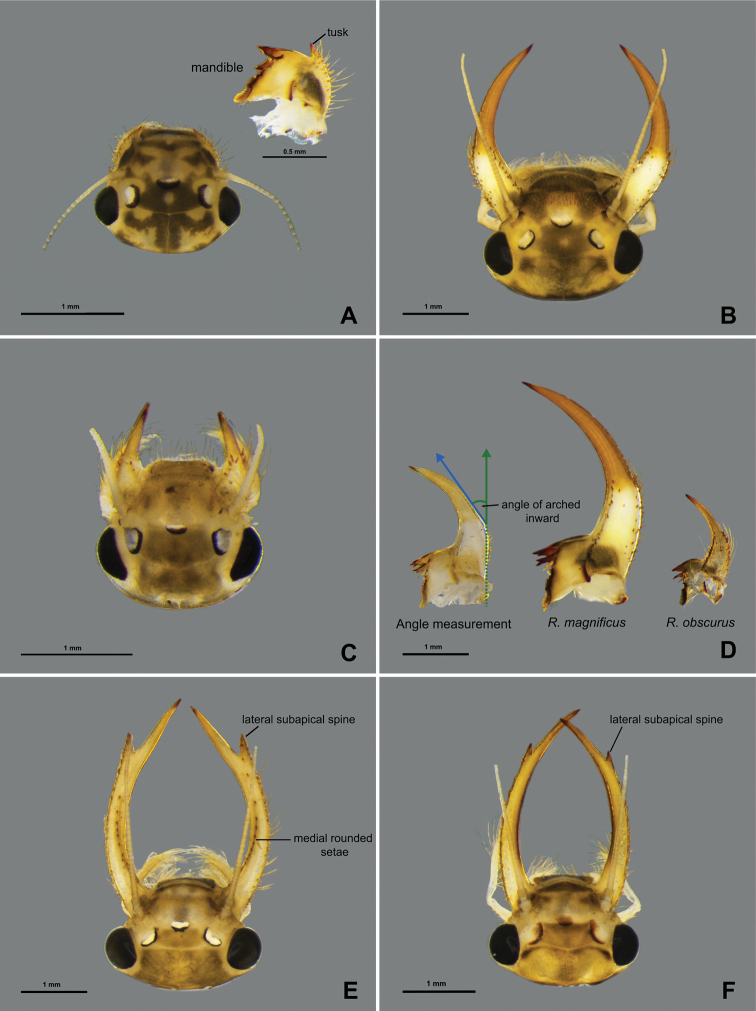
Larval morphology (head and mandibular tusk) **A***Potamanthusformosus* Eaton, 1892 **B***Rhoenanthusmagnificus* Ulmer, 1920 **C***R.obscurus* Navás, 1922 **D** angle measurement of mandibular tusk **E***R.distafurcus* Bae & McCafferty, 1991 **F***R.speciosus* Eaton, 1881.

**Figure 11.**
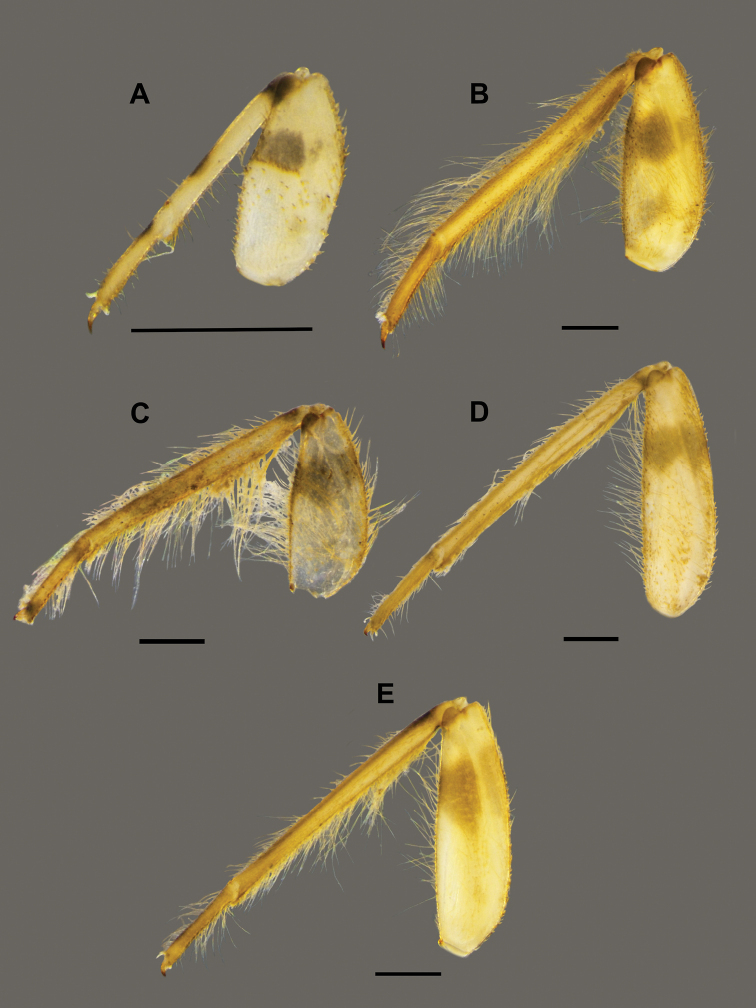
Larval morphology (foreleg) **A***Potamanthusformosus* Eaton, 1892 **B***Rhoenanthusmagnificus* Ulmer, 1920 **C***R.obscurus* Navás, 1922 **D***R.distafurcus* Bae & McCafferty, 1991 **E***R.speciosus* Eaton, 1881. Scale bar: 1 mm.

**Figure 12.**
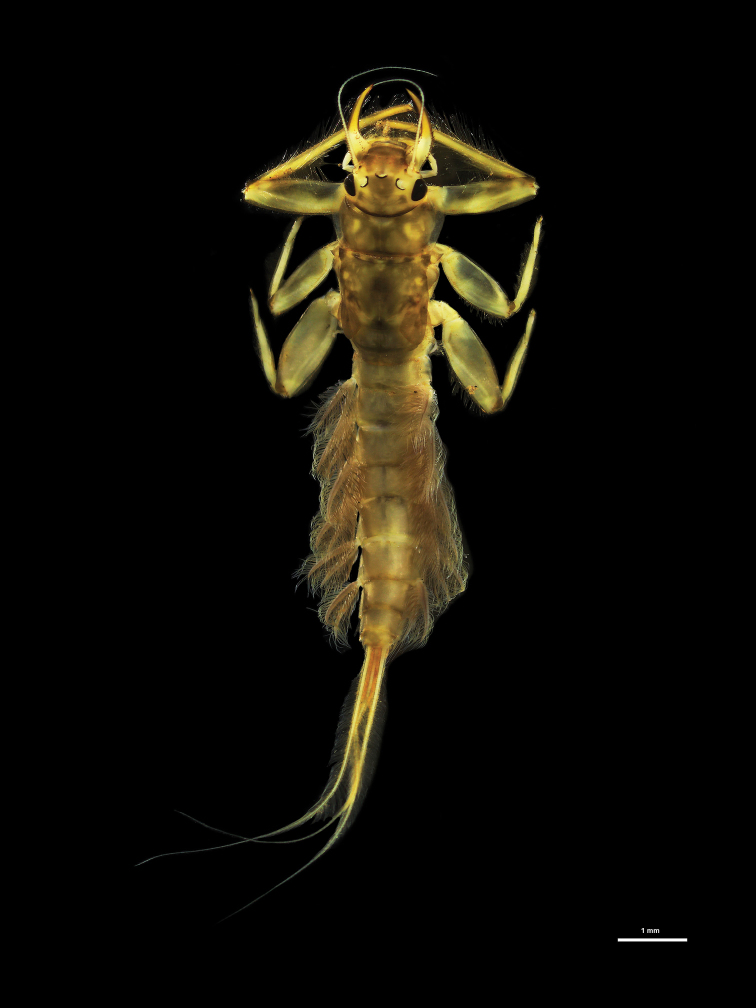
Habitus of larva of *Rhoenanthusmagnificus* Ulmer, 1920.

**Figure 13.**
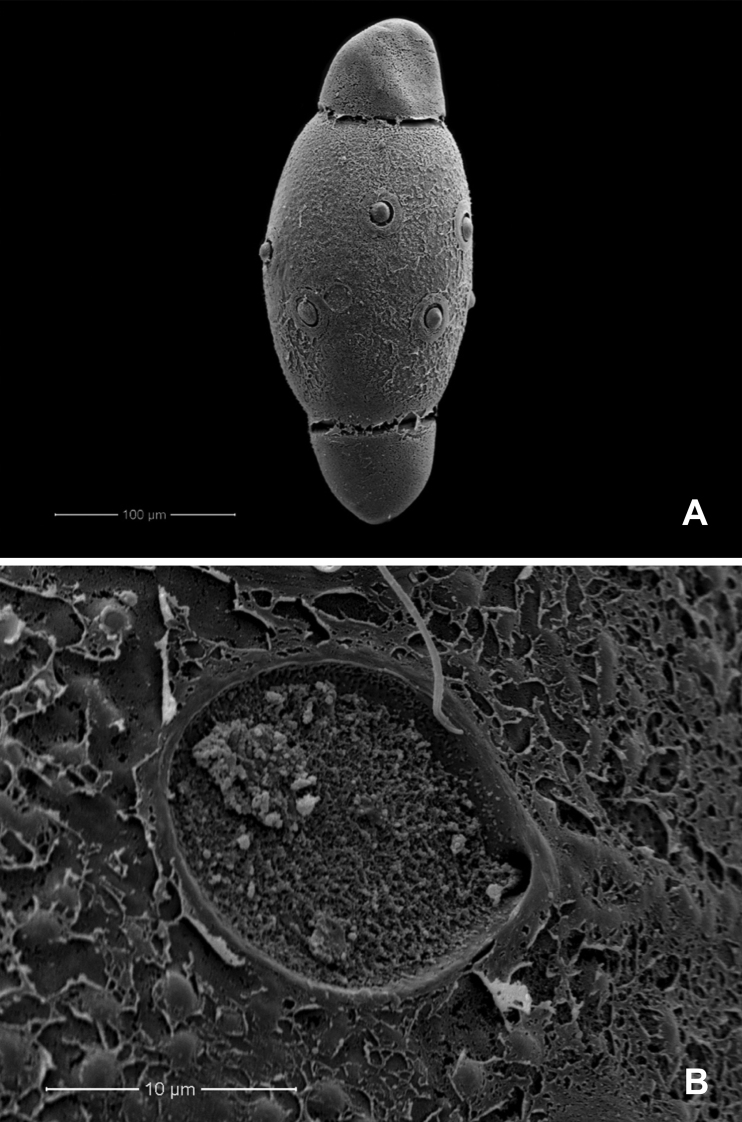
SEMs of egg structures of *Rhoenanthusspeciosus* Eaton, 1881 **A** general outline of egg **B** micropyle.

### Genus *Rhoenanthus* Eaton, 1881

**SubgenusPotamanthindus Lestage, 1930**

#### Rhoenanthus (Potamanthindus) magnificus

Taxon classificationAnimaliaEphemeropteraPotamanthidae

Ulmer, 1920

3BD887F4-9F02-5DB6-8BA8-297F43999C37

**Figure 14.**
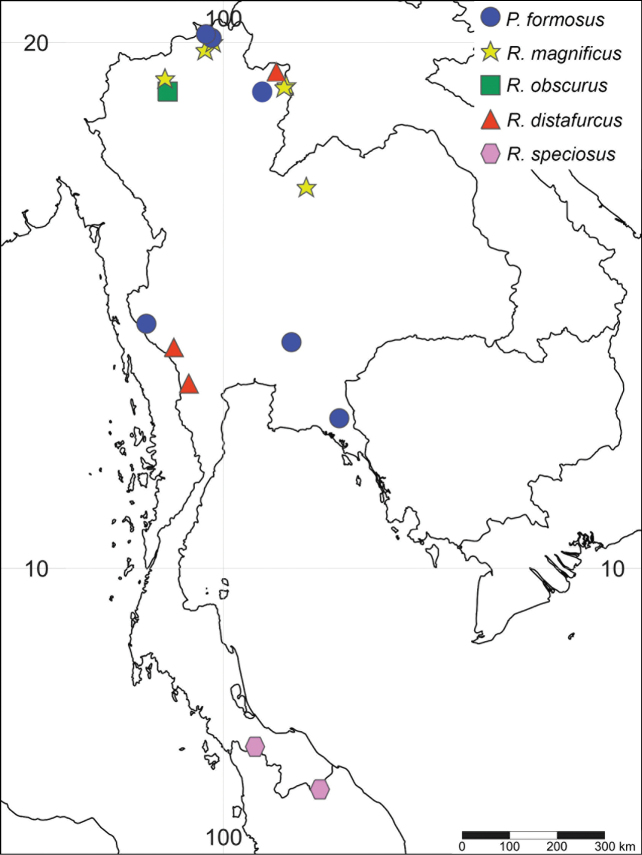
Distribution map of the family Potamanthidae in Thailand.

**Figure 15.**
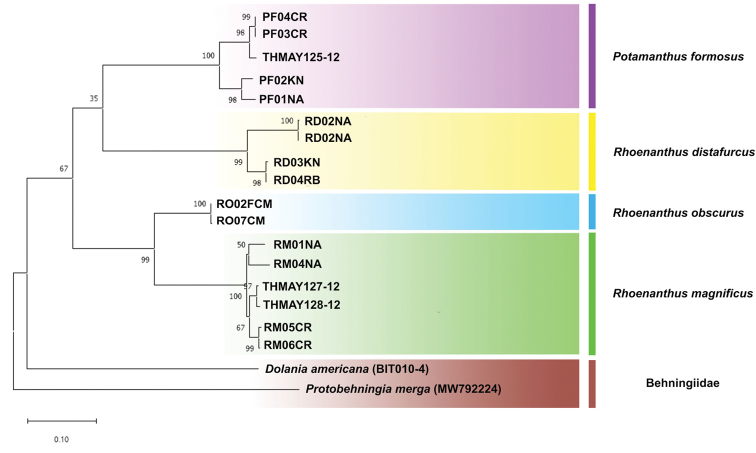
The COI phylogenetic construction based on the maximum likelihood (ML) analysis of family Potamanthidae in Thailand.

Figures 10B, D , 11B , 12 , 14 , 15

##### Materials examined.

5 larvae, Thailand, Chiang Mai province, Chiang Dao, Mae Na, 19°19'13.08"N, 98°53'25.98"E, 742 m, 11.III.2016, B. Boonsoong leg. (ZMKU); 8 larvae, Loel province, Nam Thob ranger station, 17°15'36.5"N, 101°34'52.9"E, 338 m, 20.III.2016, B. Boonsoong leg. (ZMKU); 1 larva, Nan province, Bo Kluea district, Sapan waterfall, 19°11'25.8"N, 101°11'56.3"E, 800 m, 28.XI.2020, B. Boonsoong leg; 2 larvae, Nan province, Bo Kluea district, Lamer resort, 19°09'08.8"N, 101°09'17.0"E, 28.XI.2020, S. Kwanboon leg; 3 larvae, Nan province, Bo Kluea district, Mae Nam Wa stream, 19°16'22.6" N 101°10'48.2" E, 848 m, 26.XI.2019, B. Boonsoong leg; 7 larvae, Chiang Rai province, Mueang district, Mae Kon stream, 19°51'46.1"N, 99°39'04.7"E, 534 m, 6.III.2021, S. Kwanboon leg; 2 larvae, Chiang Rai province, Mueang district, Mae Kon stream, Pong Phrabat waterfall, 20°00'41.8"N, 99°48'15.1"E, 470 m, 7.III.2021, S. Kwanboon leg.

##### Diagnosis.

The larvae of *Rhoenanthusmagnificus* (Fig. 12) can be distinguished from those of other Rhoenanthus (Potamanthindus) species based on the following characteristics: i) large body size (18–21 mm), ii) mandibular tusks arched inward about 33–34° (angle measurement as shown in Fig. 10D), iii) length of the mandibular tusks ca 1.4× length of head, and iv) length of the foretibiae ca 1.5× length of the forefemora and about 2.9× length of the foretarsi (Fig. 11B) (Nguyen and Bae 2004).

##### Distribution.

Chiang Mai, Chiang Rai, Loei, and Nan provinces.

##### Remark.

The larva of *R.magnificus* was originally described by Nguyen and Bae (2004) from material collected in northern and central Vietnam. The species is known from southern China and Vietnam. In the present study, we found this species in streams of several provinces (Fig. 14).

#### Rhoenanthus (Potamanthindus) obscurus

Taxon classificationAnimaliaEphemeropteraPotamanthidae

Navás, 1922

C0F8D3C4-3C5B-57E1-9004-14B00792A55C

Figures 10C, D , 11C , 14 , 15

##### Materials examined.

1 female imago (reared) and 1 male imago (reared), Thailand, Chiang Mai province, Mae Ping river, Elely Cafe, 19°04'08.4"N, 98°56'28.8"E, 15.XI.2020, S. Kwanboon leg. (ZMKU).

##### Diagnosis.

The larvae of *Rhoenanthusobscurus* can be distinguished from those of other Rhoenanthus (Potamanthindus) species based on the following characteristics: i) medium-sized body (12–17 mm), ii) mandibular tusks arched inward about 28° (angle measurement as shown in Fig. 10D), iii) length of mandibular tusks ca 0.7–0.8× length of the head, and iv) length of foretibiae ca 1.32–1.49× length of the forefemora and about 2.55–3.02× length of the foretarsi (Fig. 11C) (Bae and McCafferty 1991; Nguyen and Bae 2006).

##### Distribution.

Chiang Mai province.

##### Remark.

The larva of *R.obscurus* was originally described by Gose (1969) as *Potamanthus* sp. TPA and collected from Thailand (Chantaburi province). Bae and McCafferty (1991) redescribed the larva with material from Mae Ping river, Chiang Mai province. In this study, we found this species in the same river as in the previous study. Our specimens were reared in the laboratory and successfully raised to the imago stage.

### Subgenus Rhoenanthus Eaton, 1881

#### Rhoenanthus (Rhoenanthus) distafurcus

Taxon classificationAnimaliaEphemeropteraPotamanthidae

Bae & McCafferty, 1991

A6C559C9-16A6-5668-9E55-070F4371094B

Figures 10E , 11D , 14 , 15

##### Materials examined.

1 larva, Thailand, Kanchanaburi province, Sai Yok district, Pueng Wahn Resort, 14°12'08.9"N, 99°03'36.0"E, 15.X.2015, B. Boonsoong leg; 2 larvae, Ratchaburi province, Suan Phueng district, Pha Chi river, 13°30'57.3"N, 99°20'40.1"E, 19.IX.2016, B. Boonsoong leg; 1 larva, Nan province, Bo Kluea district, Sapan waterfall, 19°11'25.8"N, 101°11'56.3”E, 800 m, 28.XI.2020, B. Boonsoong leg.

##### Diagnosis.

The larvae of *Rhoennanthusdistafurcus* can be distinguished from those of other Rhoenanthus (Rhoenanthus) species based on the following characteristics: i) subapical spine of the mandibular tusk well developed laterally (Fig. 10E), with a simple, short spine, ii) 16–20 medial rounded setae on mandibular tusk (Fig. 10E) iii) length of the mandibular tusks ca 1.7–1.9× length of head, iv) length of foretibiae ca 1.19–1.25× length of the forefemora and about 2.5–2.8× length of the foretarsi, (v) leg with colour marking as in Fig. 11D, and vi) lack of bipectinated setae on the mandible (Soldan T and Putz M 2000).

##### Distribution.

Kanchanaburi, Ratchaburi, and Nan provinces.

##### Remark.

Bae and McCafferty (1991) described *R.distafurcus* based on imaginal specimens from Thailand, India, and Vietnam. The larva of *R.distafurcus* was described by Soldan T and Putz M (2000) based on specimens from Vietnam. In Thailand, a male adult of this species was found in Khao Yai National Park (Bae and McCafferty 1991). In the present study, larval specimens of this species were found in western and northern Thailand.

#### Rhoenanthus (Rhoenanthus) speciosus

Taxon classificationAnimaliaEphemeropteraPotamanthidae

Eaton, 1881

BB3B5E97-D40A-5257-B3E9-46F0D0CDDEE5

Figures 10F , 11E , 13 , 14

##### Materials examined.

5 larvae, Thailand, Narathiwat province, Klong Aika Ding stream, 5°47'45.9"N, 101°50'05.5"E, 22.IV.2018, B. Boonsoong leg.

##### Diagnosis.

The larvae of *Rhoenanthusspeciosus* can be distinguished from those of other Rhoenanthus (Rhoenanthus) species based on the following characteristics: i) lateral subapical spine of the mandibular tusk present (Fig. 10F), ii) absence of medial rounded setae on the mandibular tusk, iii) length of the mandibular tusks ca 1.7–2.3× length of the head, iv) length of the foretibiae ca 1.2–1.23× length of the forefemora and about 2.72–2.82× length of the foretarsi, v) leg with colour marking as in Fig. 11E, and vi) 4 or 5 bipectinated lateral setae on the mandibles (Bae and McCafferty 1991).

**Egg** (dissected from mature larva). Oval; with two large conical polar caps, (Fig. 13A); chorion with numerous scattered tubercles, with knob-terminated coiled threads at the equatorial zone; tagenoform micropyle; sperm guide circular (Fig. 13B).

##### Distribution.

Narathiwat and Songkla provinces.

##### Remark.

The larvae of *Rhoenanthusspeciosus* were reported by Bae and McCafferty (1991) based on specimens from Indonesia and Malaysia. Parnrong et al. (2002) reported this species from Songkhla province (southern Thailand). In the present study, we found the larva of this species in the nearby Narathiwat province. The distribution of *R.speciosus* seems to be restricted to the south of the Isthmus of Kra, as was found for another mayfly species, *Prosopistomawouterae* (Boonsoong & Sartori, 2019). These findings constitute the northern limit of the known distribution of this species.

### Key to genera and species of Potamanthidae in Thailand

(adapted from Bae and McCafferty 1991; Nguyen and Bae 2006)

**Table:** 

1	Mandibular tusks subequal to, or longer than 1/2 length of head (Fig. 10C, E, F)	***Rhoenanthus*, 2**
–	Mandibular tusks shorter than 1/2 length of head (Fig. 10A)	***Potamanthus*,subgenus Potamanthodes, *P.formosus***
2	Mandibular tusks with lateral subapical spine, appearing apically forked	**subgenusRhoenanthus, 3**
–	Mandibular tusks without lateral subapical spine, not appearing apically forked	**subgenusPotamanthindus, 4**
3	Mandibular tusks with large lateral subapical spine with 16–20 medial rounded setae (Fig. 10E)	***R.distafurcus***
–	Mandibular tusks with small lateral subapical spine, without medial rounded setae (Fig. 10F)	***R.speciosus***
4	Mandibular tusks strongly convergent and abruptly curved inward about 33–34° (Fig. 10D), length of the mandibular tusks ca 1.4× length of head (Fig. 10B)	***R.magnificus***
–	Mandibular tusks strongly convergent and abruptly curved inward about 28° (Fig. 10D), length of the mandibular tusks ca 0.7–0.8× length of head (Fig. 10C)	***R.obscurus***

### Molecular analysis

The partial sequence of the mitochondrial COI gene (658 bp) of *P.merga* (MW792224) found in Thailand was analysed and compared with the sequence of *Dolaniaamericana* (BIT011-04) from BOLD. However, there is no available sequence for the genus *Behningia*. The intergeneric genetic distance between these two genera was 22.39%, as determined by the Kimura 2-parameter (K2P) model. For Potamanthidae, the phylogenetic tree of the ML analysis is shown in Figure 15 and depicts four clearly separated clades delineating four species. No sequence of *Rhoenanthusspeciosus* are included due to unsuccessful DNA extraction. Analysis of the K2P genetic distance to confirm the species delimitation revealed that the intraspecific genetic distances vary between 0.2–5.4%, whereas the interspecific distances are high, ranging from 14–20% (Table 2). The lowest interspecific distance value was found between *R.magnificus* and *R.obscurus* (14%), which share close morphological characters.

**Table 2.** Pairwise genetic distances (COI) between species of Potamanthidae using the Kimura 2-parameter.

		1	2	3	4
**1**	*Potamanthusformosus*	–			
**2**	*Rhoenanthusobscurus*	0.171–0.190	–		
**3**	*Rhoenanthusmagnificus*	0.181–0.212	0.125–0.158	–	
**4**	*Rhoenanthusdistafurcus*	0.177–0.198	0.171–0.196	0.182–0.210	–

## Discussion

The discovery of an additional species of *Behningia* in Thailand reveals the high diversity of the behningiid mayflies in the country. The presence of *B.nujiangensis* was confirmed based on morphological evidence according to Zhou et al. (2019) and McCafferty and Jacobus (2006). In the present study, larvae of *B.nujiangensis* were collected from a stream in Chiang Mai province, whereas Zhou et al. (2019) described the species from the Nujiang river (China, upper Salween river), a short section of the river that flows through northern Thailand (Fig. 9). The habitat of *B.nujiangensis* is restricted to sandy bottoms in streams or rivers. The larval exuviae and imagoes of *P.merga* were known only from a river in Kanchanaburi province (western Thailand) by Peters and Gillies (1991). In addition, larvae of *P.merga* were collected from the Mae Chaem river, Chiang Mai province (northern Thailand). This a second report and a new distribution record for this species. The eggs of the genus *Behningia* are the largest known among mayflies, with *B.nujiangensis* reaching more than 1 mm in length. The length of eggs of *Behningialestagei* (0.9–1 mm) and *Dolaniaamericana* (0.7–0.8 mm) were reported by Keffermuller (1959) and Koss and Edmunds (1974). The egg structure of *B.nujiangensis* is similar to that of *Behningialestagei*. The position of adhesive material differs between *Behningia* and *Dolania*. However, the egg of *Protobehningia* is still unknown.

The presence of *R.magnificus* in Thailand was confirmed based on the morphological characters proposed by Nguyen and Bae (2004). This species is a new record in Thailand. In this study, the larvae of *R.magnificus* were collected from streams and rivers, where they were often found at the interface of small stones and finer substrate (sand and gravel) in the slow current streams, as previously reported by Nguyen and Bae (2004). Our results allow us to conclude that five valid species of the family Potamanthidae exist in Thailand, as supported by morphological and molecular analyses.

## Conservation issues of the Behningiidae

The larvae of Behningiidae are restricted to fine sandy habitats (Peters and Gillies 1991; McCafferty and Jacobus 2006; Park et al. 2019; Zhou et al. 2019). The habitat of *Behningiatshernovae* Edmunds & Traver, 1959 in Korea is restricted to fine sand streams, and high-quality water is needed for its survival (Park et al. 2019). *Behningiaulmeri* is a very rare and extremely endangered European lowland species. In Poland, it may have become extinct as well, and any protective measures there would seem useless (Bauernfeind and Soldán 2012). Among the Thai behningiid mayflies, *B.baei* was found in the Klong Namkub (Phitsanoluk province) in 2002 and *B.nujiangensis* in the Tard Luang waterfall (Chiang Mai province) in 2011, whereas *Protobehningiamerga* was found in the Khwae Noi river (Kanchanaburi province) in 1987 and has not been found again in re-samplings. Thai streams and rivers are altered by channel alterations, dam constructions, and sand harvesting. The sandy habitats have gradually decreased in Thailand, and this has threatened the survival of sand-dwelling organisms, including behningiid mayflies. The conservation of fine sandy habitat is, therefore, required to protect this extremely specialized psammophilous fauna.

## Supplementary Material

XML Treatment for Behningia baei

XML Treatment for Behningia nujiangensis

XML Treatment for Protobehningia merga

XML Treatment for Potamanthus (Potamanthodes) formosus

XML Treatment for Rhoenanthus (Potamanthindus) magnificus

XML Treatment for Rhoenanthus (Potamanthindus) obscurus

XML Treatment for Rhoenanthus (Rhoenanthus) distafurcus

XML Treatment for Rhoenanthus (Rhoenanthus) speciosus
